# Agonist bias and agonist‐dependent antagonism at corticotrophin releasing factor receptors

**DOI:** 10.1002/prp2.595

**Published:** 2020-06-11

**Authors:** Zoe Tasma, Peter Wills, Debbie L. Hay, Christopher S. Walker

**Affiliations:** ^1^ School of Biological Sciences University of Auckland Auckland New Zealand; ^2^ Maurice Wilkins Centre and Centre for Brain Research University of Auckland Auckland New Zealand

**Keywords:** corticotropin releasing factor, CRF_1_, CRF_2_, functional selectivity, intracellular signaling, probe‐dependent antagonism

## Abstract

The corticotropin‐releasing factor (CRF) receptors represent potential drug targets for the treatment of anxiety, stress, and other disorders. However, it is not known if endogenous CRF receptor agonists display biased signaling, how effective CRF receptor antagonists are at blocking different agonists and signaling pathways or how receptor activity‐modifying proteins (RAMPs) effect these processes. This study aimed to address this by investigating agonist and antagonist action at CRF_1_ and CRF_2_ receptors. We used CRF_1_ and CRF_2_ receptor transfected Cos7 cells to assess the ability of CRF and urocortin (UCN) peptides to activate cAMP, inositol monophosphate (IP_1_), and extracellular signal‐regulated kinase 1/2 signaling and determined the ability of antagonists to block agonist‐stimulated cAMP and IP_1_ accumulation. The ability of RAMPs to interact with CRF receptors was also examined. At the CRF_1_ receptor, CRF and UCN1 activated signaling in the same manner. However, at the CRF_2_ receptor, UCN1 and UCN2 displayed similar signaling profiles, whereas CRF and UCN3 displayed bias away from IP_1_ accumulation over cAMP. The antagonist potency was dependent on the receptor, agonist, and signaling pathway. CRF_1_ and CRF_2_ receptors had no effect on RAMP1 or RAMP2 surface expression. The presence of biased agonism and agonist‐dependent antagonism at the CRF receptors offers new avenues for developing drugs tailored to activate a specific signaling pathway or block a specific agonist. Our findings suggest that the already complex CRF receptor pharmacology may be underappreciated and requires further investigation.

**Abbreviations**
CLR: calcitonin receptor‐like receptor
CRF: corticotropin releasing factor
CTR: calcitonin receptor
ERK1/2: extracellular signal‐regulated kinase 1/2
GPCR: G protein‐coupled receptor
IP_1_: inositol monophosphate
RAMP: receptor activity‐modifying protein
UCN: urocortin

## 1 INTRODUCTION

The neuropeptide, corticotropin releasing factor (CRF) is a member of the secretin peptide family.^1^, ^2^ CRF is expressed throughout the central nervous system and in peripheral tissues.^3^ CRF is closely related to the three urocortin (UCN) peptides, which share a similar structure, and have overlapping receptors and functions.^4^ The expression of the three UCN peptides are less well‐characterized than CRF, however, they are expressed at both overlapping and discrete sites within the CNS.^5^, ^6^

CRF is best characterized for its roles in stress and anxiety.^5^ In the pituitary, CRF stimulates the release of adrenocorticotropic hormone (ACTH). ACTH subsequently elevates circulating glucocorticoid steroids, which allow an organism to respond to stressful situations.^5^, ^7^ Elevated CRF concentrations have also been observed in a number of psychiatric disorders, indicating that drugs targeting this system could have utility in treating stress and anxiety.^7^ The UCN peptides have also been implicated in stress responses; potentially regulating the recovery from stressful stimuli and ACTH release from the pituitary.^8^, ^9^, ^10^ The UCN peptides have additional functions centrally, including modulating social behaviors, and peripherally, such as the modulation of vasodilation, cardiac output, and the control of metabolism.^11^, ^12^, ^13^, ^14^, ^15^ This suggests that drugs targeting the UCN axis could have added utility in treating other behavioral disorders, cardiovascular disease, obesity, and diabetes.

The CRF peptide family can bind two G protein‐coupled receptors (GPCRs); CRF_1_ and CRF_2_. The CRF_1_ receptor binds CRF and UCN1 with high affinity, whereas the CRF_2_ receptor can bind CRF, UCN1, UCN2, and UCN3 with high affinity.^16^, ^17^ The CRF receptors have already been exploited in drug discovery resulting in small molecule CRF_1_ antagonists which have been explored in clinical trials to treat anxiety, depression and addiction.^18^ Several CRF_1_ receptor antagonists are reportedly safe and clinical trials are ongoing, whereas other CRF receptor antagonists have been discontinued due to a lack of efficacy.^18^ The underlying basis for these differences is not well understood but it is possible that CRF receptor pharmacology is more complicated than presently appreciated. For example, both the CRF_1_ and CRF_2_ receptors have functional splice variants and have been reported to form heterodimers with a receptor activity‐modifying protein (RAMP).^19^, ^20^, ^21^ However, there are conflicting reports for RAMP interactions.^22^ RAMPs can form heterodimers with several GPCRs altering cell surface expression, receptor trafficking, pharmacology, and/or signaling properties.^23^

The activation or specific inhibition of a discrete signaling pathway can be associated with a biological event, driven by biased ligands. Such molecules can activate the same receptor, giving rise to a different pattern of signaling and potentially different biological outcomes.^1^, ^24^ This has led to considerable interest in exploring biased signaling for both endogenous ligands and in drug discovery, where the goal is to activate or block a specific pathway.^25^, ^26^ Exploiting the therapeutic potential of the UCN peptides also relies on a greater understanding of how these peptides trigger CRF receptor signaling.

In order to enable further exploitation of CRF receptor‐mediated signaling pathways in drug discovery, we profiled several signaling pathways in response to the endogenous peptides CRF and the three UCN peptides. Moreover we investigated how effectively CRF receptor antagonists block CRF‐ or UCN1‐stimulated receptor signaling.

## 2 METHODS

### 2.1 Peptides and antagonists

Human CRF (CRF) and human urocortin 1 (UCN1) were purchased from the American Peptide Company or Bachem. Human urocortin 2 (UCN2), human urocortin 3 (UCN3), α‐helical CRF_(9‐41)_, and astressin_2B_ were purchased from the American Peptide Company. CP‐376,395 was purchased from Tocris Bioscience.

### 2.2 Plasmid constructs

Plasmids containing hCRF_1α_ and hCRF_2α_ (GenBank accession numbers AY457172 and AY449734) were obtained from the cDNA Resource Centre (Bloomsburg University) and are referred to as CRF_1_ and CRF_2_ in this manuscript. Hemagglutinin (HA) epitope‐tagged human Calcitonin (CT) receptor (CT_(a)_ splice variant) and myc‐tagged human RAMP1 were used as described previously.^27^ HA‐tagged human calcitonin receptor‐like receptor (CLR), N‐terminal FLAG‐tagged human RAMP3 and N‐terminal myc‐tagged human RAMP3 were a gift from Professor David Poyner (Aston University) and Professor Patrick Sexton (Monash University), respectively. N‐terminal FLAG‐tagged human RAMP2 was used as described previously.^28^ All receptors and RAMPs were cloned in pcDNA3.0 or pcDNA3.1 plasmid vectors. All plasmid inserts were sequenced at the Centre for Genomics, Proteomics and Metabolomics (University of Auckland) and the sequences were verified prior to use.

### 2.3 Cell culture and transfection

Cos7, HEK‐293S, and HEK‐293T cells were cultured as described previously.^28^, ^29^ Cos7 cells were originally supplied by the ATCC to Associate Professor Nigel Birch (University of Auckland). HEK‐293S cells were a gift from Professor David Poyner (Aston University) and HEK‐293T cells a gift from Professor John Taylor (University of Auckland). Briefly, all cell lines were cultured in Dulbecco's Modified Eagle Medium (DMEM) supplemented with 8% heat inactivated fetal bovine serum (FBS) and maintained in a humidified incubator at 37°C with 5% CO_2_. For transfection, cells were counted (Countess CounterTM; Life Technologies) and plated at a density of 20 000 cells per well in 96‐well plates. The cells were transiently transfected 48 hours prior to assaying using polyethylenimine (PEI), as described previously.^28^, ^29^ In all experiments a total of 0.25 μg DNA was transfected per well. In the majority of experiments 0.25 μg DNA of receptor‐containing plasmid or the pcDNA3.1 plasmid without an insert was used. For experiments involving RAMPs, 0.125 μg of receptor DNA and 0.125 μg of RAMP‐containing plasmid DNA were transfected to give a 1:1 ratio of receptor to RAMP. Where the RAMP or receptor were transfected alone, the ratio of DNA was made up with the pcDNA3.1 plasmid.

### 2.4 cAMP measurement in Cos7 cells

cAMP accumulation in Cos7 cells transfected with hCRF_1α_ or hCRF_2α_ receptors was measured using the LANCE cAMP detection assay kit (PerkinElmer Life and Analytical Sciences) as described previously.^30^ Briefly, culture media was removed and replaced with DMEM containing 0.1% bovine albumin serum (BSA) and 1 mmol/L 3‐isobutyl‐1‐methylxanthine (IBMX) for 30 minutes at 37°C. Cells were incubated for an additional 15 minutes at 37°C with media or agonist concentrations of 1 pmol/L to 1 µmol/L for agonist only assays or 10 pmol/L to 10 µmol/L concentrations for antagonist experiments. Antagonists were added simultaneously or immediately prior to the addition of agonists. To stop cell stimulation and extract cAMP, the contents of the wells were replaced with 50 µL absolute ethanol. Samples were left at −20°C for a minimum of 10 minutes. The ethanol was evaporated and replaced with 50 µL of cAMP detection buffer. Samples were shaken for 15 minutes before 5 µL cell lysate was transferred to a white 384‐well optiplate and processed for cAMP quantification as described previously.^31^ Samples were read using an Envision plate reader (PerkinElmer). cAMP concentrations were determined from a standard curve generated in duplicate.

### 2.5 Measurement of extracellular signal‐regulated kinase 1/2 phosphorylation in Cos7 cells

The AlphaLISA Surefire Ultra extracellular signal‐regulated kinase 1/2 (ERK1/2) phosphorylation (pERK1/2) assay kit (PerkinElmer Life and Analytical Sciences) was used to measure pERK1/2 in cell lysates after agonist stimulation. For these assays 20% FBS was used as a positive control. Following peptide stimulation at 37°C for 0‐30 minutes with 100 nmol/L agonist (time courses) or 7 minutes with media alone or 1 pmol/L to 1 µmol/L agonist, media was removed and 20 µL of lysis buffer added to each well. Plates were shaken at room temperature for 10 minutes. Each sample was then transferred to a 384‐well optiplate and pERK1/2 measurement performed as per the manufacturer's instructions. Samples were read using an Envision plate reader (PerkinElmer).

### 2.6 IP_1_ measurement in Cos7 and HEK‐293S cells

The IP‐one Gq assay kit (Cisbio) was used to quantify accumulated myo‐inositol‐1‐phosphate (IP_1_), a by‐product of IP_3_ produced after receptor‐mediated Gαq activation in Cos7 and HEK‐293S cells. Briefly, culture media was removed and replaced with DMEM containing 0.1% BSA for 30 minutes at 37°C. Cells were then incubated with media containing 50 mmol/L lithium chloride (LiCl) for an additional 0‐120 minutes (time courses) or 90 minutes at 37°C in the presence or absence of an antagonist. Agonist concentrations of 1 pmol/L to 10 µmol/L were used for agonist only assays or 10 pmol/L to 10 µmol/L concentrations for antagonist experiments. After cell stimulation, 14 µL of stimulation buffer was added to each well. Three microlitres of each detection antibody were added in turn to the plate and incubated at room temperature for 1 hour. Fifteen microliters of sample was then transferred to a 384‐well optiplate and read by an Envision plate reader (PerkinElmer). IP_1_ concentrations were calculated from a standard curve generated in duplicate.

### 2.7 Measurement of RAMP cell surface expression by ELISA

Cos7, HEK‐293T or HEK‐293S cells were plated into 96‐well plates, transfected and assayed for cell‐surface expression as described previously.^32^ Briefly, transfected cells were fixed using 4% paraformaldehyde in PBS for 20 minutes, then washed twice with PBS. One hundred microliters of PBS was added to each well and the plates stored at 4°C until further analyzed. The PBS was aspirated, and the cells incubated at room temperature with 0.6% hydrogen peroxide in PBS for 20 minutes. Cells were washed twice in PBS and blocked with 10% goat serum/PBS for 1 hour at room temperature. Cells were incubated at 37°C for 30 minutes with anti‐myc (1:250; Calbiochem EMD Biosciences) or anti‐FLAG (1:1000; Sigma Aldrich) diluted in 1% goat serum/PBS. After washing with PBS, anti‐mouse HRP (1:500; GE Healthcare Amersham) diluted in 1% goat serum/PBS was added and incubated for 1 hour at room temperature. Cells were washed twice with PBS. Fifty microliters of Sigma FAST OPD was added to each well and incubated in the dark for 15 minutes before addition of 0.5 mol/L sulfuric acid. Absorbance in samples was measured using an Envision plate reader (PerkinElmer) before and after staining with 1% cresyl violet solution to control for cell density.

### 2.8 Experimental design and data analysis

In all experiments the position of peptides and antagonists (pharmacology) or transfected receptors (ELISAs) on assay plates was randomized during each independent experiment, which are independent biological replicates. In all cases, duplicate, triplicate, or quadruplicate technical replicates were conducted for each independent experiment. For transfected cell experiments, each independent experiment involved plating cells from a distinct passage, separate transient transfection and separate signaling or ELISA assays, constituting experimental n. For ERK1/2 and IP_1_ signaling pathways, time‐course experiments were first conducted with a saturating concentration of UCN1 to determine the optimal time to conduct concentration‐response experiments. Concentration‐response experiments were then conducted with the same experimental design for cAMP, ERK1/2, and IP_1_ pathways. For signaling assays, the relevant control peptide (CRF or UCN1) was included on each assay plate in each independent experiment. The requirement for multiple concentrations of agonist/antagonist to be made‐up by a single operator for individual assays resulted in blinding not being feasible. All group sizes were designed to be equal at n = 5 independent experiments. However, when *F* tests performed on individual experiments indicated that a single curve could fit to both agonist and antagonist curves or no agonist concentration‐response curve could be fitted to the data, neither p*K_B_* nor pEC_50_ values could be determined, respectively. Therefore, no statistical comparisons were performed and experiments were curtailed at n = 3‐4 individual experiments. For antagonism of UCN1‐mediated IP_1_ accumulation by CP‐376,395 at the CRF_2_ receptor, one additional experiment was performed. All data were plotted and analyzed using GraphPad Prism 6.0 or 7.0 (GraphPad Software Inc). Data points are the mean ± standard error of the mean (SEM) from n separate experiments, combined.

### 2.9 Agonist assays

For agonist signaling assays data were fitted with a four‐parameter logistic equation. *F* tests were performed to determine if the Hill slope was significantly from one (GraphPad Prism). When the Hill slope was not significantly different from one the curves were constrained to one and pEC_50_ values obtained. When the Hill slope was significantly different from one, this parameter was unconstrained. To combine the data, maximal responses (*E*_max_) were determined and the data expressed as a percentage of the *E*_max_ obtained for matched UCN1 on the same assay plate. For pERK1/2 time course assays, the data were normalized to the response from 20% FBS conducted in parallel. Data normalization was necessary due to variation introduced by transient receptor transfection.

### 2.10 Signaling bias

Agonist signaling bias was calculated as published previously.^33^ UCN1 acted as a full agonist at all signaling pathways examined and was used as the reference ligand. Briefly, using equations for the *Operational Model for Bias*, GraphPad Prism 7.0 was used to determine Log(*τ*/*K*_A_). The Hill slope was determined using the procedure outlined for agonist assays. Thus, the Hill slope was constrained to one for cAMP and IP_1_ assays and unconstrained for pERK1/2, the *E*_max_ parameter was shared or set to 100 if the fit was ambiguous, to reflect the reference ligand response. ΔLog(*τ*/*K*_A_) was then calculated by subtracting the reference ligand Log(*τ*/*K*_A_) from each test ligand Log(*τ*/*K*_a_). ΔΔLog(*τ*/*K*_A_) ratios were determined by comparing the ΔLog(*τ*/*K*_A_) for each signaling pathway to the reference signaling pathway (cAMP). The bias factor for each ligand was defined as the inverse log of the ΔΔLog(*τ*/*K*_A_) for a given ligand.

### 2.11 Antagonist assays

For antagonist assays, p*K_B_* antagonist potency values were calculated using pEC_50_ values from concentration response curves of agonist alone, or agonist in the presence of one or three different antagonist concentrations. Initially, *F* tests were performed to determine if both the agonist alone and agonist in the presence of antagonist data sets could be fitted using a single curve. When a single curve did not fit all data sets, p*K_B_* values were calculated. When the *E*_max_ in the presence of antagonist was not significantly different from the agonist alone curve (*F* test), the data were analyzed using global Schild analysis for competitive antagonists (Graphpad Prism). *F* tests were then performed to determine if the Schild slope was significantly from one. When the Schild slope was not significantly different from one, this parameter was constrained to one and antagonist p*K_B_* values were obtained. When the *E*_max_ in the presence of antagonist was significantly different from the agonist alone curve (*F* test), the method of Gaddum for an insurmountable or non‐competitive antagonist was used to determine antagonist potency.^34^ To generate curves, data points were simulated based on the equation for three parameter logistic fits. Data points between the EC_25_ and EC_75_ for antagonist curves were plotted on a double reciprocal plot to create a linear regression. The resulting slope was then used to calculate the antagonist *K_B_* when substituted into the equation *K_B_* = [*B*]/(slope − 1) (equation 6.34^34^). At the CRF_1_ receptor, CP 376‐395 data sets were further analyzed by fitting the operational model of allosterism to the combined data sets.^35^ No intrinsic activity of CP‐376,395 was observed, therefore τ_B_ was constrained to 0. The *E*_max_ was constrained to the maximum % value in the control (agonist alone) curve and E_min_ was set to 0%. When the Hill slope of the control curve was equal to one, n was set to one. All other parameters were shared between all data sets. The *β* value was constrained to 0 when initial fits reported an ambiguous value which was near 0. The CRF_2_ data sets used a single antagonist concentration and therefore could not be fitted to the operational model of allosterism.

### 2.12 ELISA assays

To compare the cell surface expression of RAMP1 and 2 between receptors, the data were normalized to the maximum surface expression generated by CLR and RAMP1 or 2 because CLR gives reproducibly high surface expression of both RAMP1 and RAMP2.^32^, ^36^ Data normalization was necessary due to variation introduced by transient receptor transfection. For FLAG‐RAMP3, normalization was not performed.

### 2.13 Statistical analysis

The data and statistical analysis comply with the recommendations on experimental design and analysis in pharmacology.^37^ All data were plotted and analyzed using GraphPad Prism 6.0 or 7.0 (GraphPad Software Inc). pEC_50_ and p*K_B_* values were averaged from separate biological replicates (individual experiments) to generate mean values. For signaling data, pEC_50_ and p*K_B_* which are log values and assumed to be normally distributed, significant differences were determined using parametric tests. When two values were compared, an un‐paired two‐tailed Student's *t* test was used. When more than two values were compared, a one‐way ANOVA with post hoc Dunnett's test was used. For cell surface expression of RAMP1 and RAMP2 (ELISAs), the mean normalized surface expression from individual experiments were combined. Significant differences were determined using one‐way ANOVA with post hoc Dunnett's test. In all cases statistical significance was defined as *P* < .05.

## 3 RESULTS

### 3.1 CRF receptors in transfected Cos7 cells exhibit biased signaling

The pharmacology of the CRF_1_ and CRF_2_ receptors was characterized in transiently transfected cells by determining the ability of their endogenous ligands to activate different intracellular signaling pathways. We selected three signaling molecules for interrogation; cAMP, IP_3_ (via IP_1_) and ERK1/2. cAMP and IP_1_ are important signaling molecules for Gαs and Gαq signaling, respectively, and pERK1/2 is reportedly important for downstream effects of CRF.^38^, ^39^ We first established that no endogenously expressed CRF‐responsive receptor was functional in Cos7 cells (Figure S1). Experiments were then performed to determine the optimal time point for pERK1/2 and IP_1_ accumulation to conduct concentration‐response experiments at CRF receptors. The data suggested that in response to CRF and UCN1, 7 minutes for pERK1/2 and 90 minutes for IP_1_ accumulation were the optimal time points for both receptors (Figure S2). Interestingly, 100 nmol/L CRF failed to stimulate IP_1_ accumulation during the time course for CRF_2_ activation (Figure S2D), suggesting that its ability to signal via this pathway is less potent than at the CRF_1_ receptor, when compared to UCN1.

**FIGURE 1:**
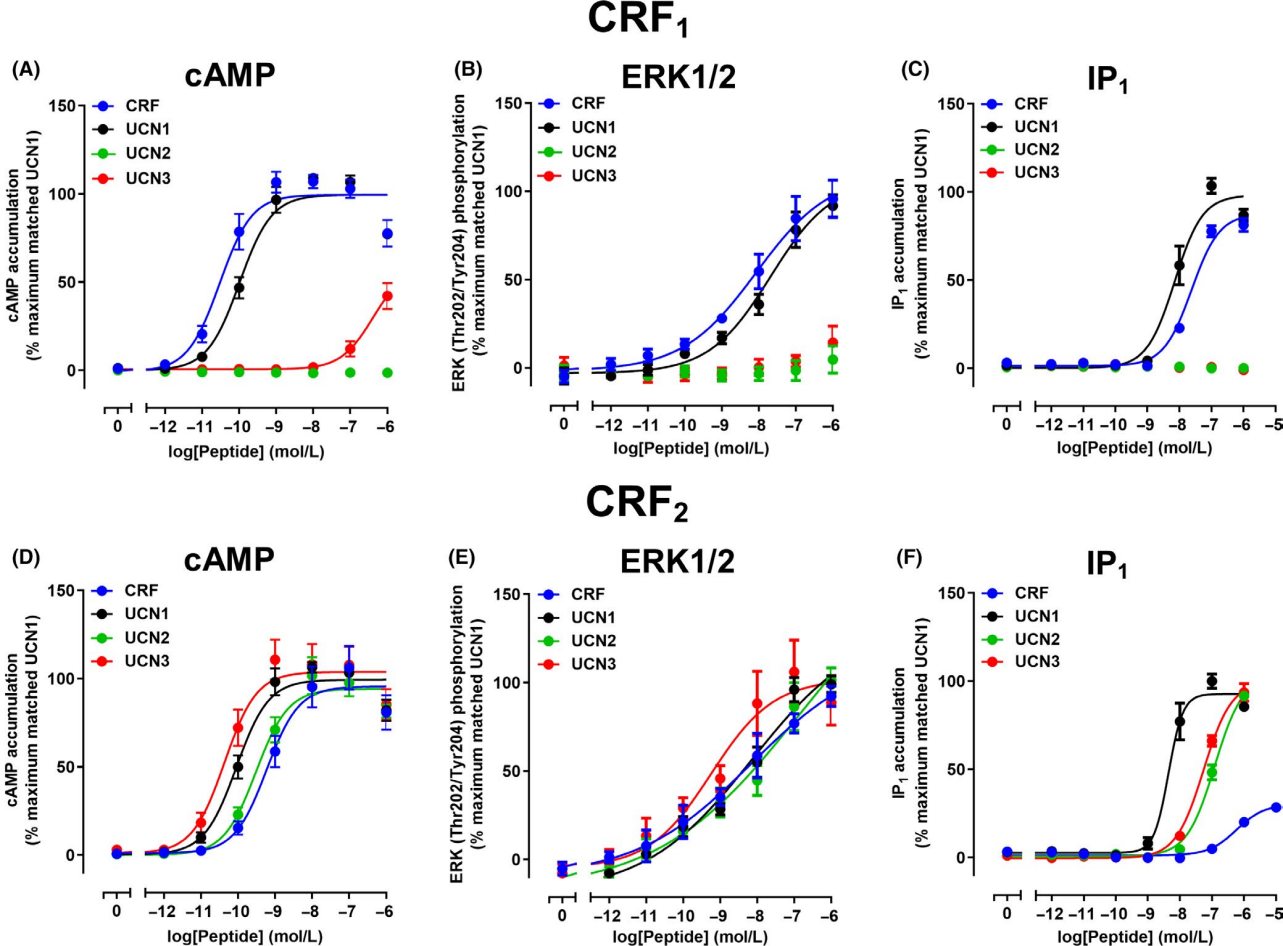
Intracellular signaling of CRF, UCN1, UCN2, and UCN3 in Cos7 cells expressing CRF_1_ or CRF_2_ receptors. A, Stimulation of cAMP accumulation by peptides at CRF_1_ receptors. B, Stimulation of ERK1/2 phosphorylation by peptides at CRF_1_ receptors. C, Stimulation of IP_1_ accumulation by peptides at CRF_1_ receptors. D, Stimulation of cAMP accumulation by peptides at CRF_2_ receptors. E, Stimulation of ERK1/2 phosphorylation by peptides at CRF_2_ receptors. F, Stimulation of IP_1_ accumulation by peptides at CRF_2_ receptors. Data points are the mean ± SEM of the combined data from five independent experiments, performed in triplicate. CRF, corticotropin releasing factor; ERK1/2, extracellular signal‐regulated kinase 1/2; IP_1_, inositol monophosphate

Concentration‐response experiments at the CRF_1_ receptor revealed that CRF and UCN1, but not UCN2 and UCN3 exhibited a concentration‐dependent increase in cAMP, pERK1/2, and IP_1_ accumulation (Figure 1A‐C; Table 1). For both cAMP and IP_1_ accumulation, the maximal responses to CRF and UCN1 were similar and the Hill slope was not significantly different from 1. For pERK1/2 the maximal responses were similar, and the Hill slope was 0.43 and 0.52 for CRF and UCN1, respectively. The relative potency between CRF and UCN1 was similar at all pathways, although CRF was significantly more potent at cAMP signaling and UCN1 was significantly more potent when IP_1_ signaling was measured. However, when comparing potency between pathways, CRF and UCN1 were approximately 100‐fold more potent at cAMP signaling than either IP_1_ or pERK1/2 (Table 1). To quantify whether UCN1 or CRF displayed a preference for a specific signaling pathway, biased signaling was assessed using the ΔΔlog(*τ*/*K*_A_) method^33^ (Table 1). This suggested that CRF was approximately 11‐fold biased for cAMP over IP_1_ accumulation relative to UCN1 at the CRF_1_ receptor.

**TABLE 1:** Summary of agonist potency (pEC_50_), relative efficacy (Δlog(*τ*/*K*_A_)) and signaling bias (ΔΔlog(*τ*/*K*_A_)) values in Cos7 cells transiently transfected with the CRF_1_ and CRF_2_ receptors

Peptide	cAMP				pERK1/2				IP_1_			
	pEC_50_	Δlog(*τ*/*K*_A_)	ΔΔlog(*τ*/*K*_A_)	n	pEC_50_	Δlog(*τ*/*K*_A_)	ΔΔlog(*τ*/*K*_A_)	n	pEC_50_	Δlog(*τ*/*K*_A_)	ΔΔlog(*τ*/*K*_A_)	n
hCRF_1α_ receptor												
CRF	10.49 ± 0.12*	0.52 ± 0.18	0.00 ± 0.26	5	7.81 ± 0.32	0.28 ± 0.51	−0.24 ± 0.58	5	7.74 ± 0.06*	−0.53 ± 0.15	−1.05 ± 0.09*	5
UCN1	9.97 ± 0.12	0.00 ± 0.17	0.00 ± 0.24	5	7.64 ± 0.19	0.00 ± 0.35	0.00 ± 0.39	5	8.11 ± 0.14	0.00 ± 0.20	0.00 ± 0.27	5
UCN2	<6	—	—	5	<6	—	—	5	<6	—	—	5
UCN3	<6	—	—	5	<6	—	—	5	<6	—	—	5
hCRF_2α_ receptor												
CRF	9.22 ± 0.07*	−0.82 ± 0.18*	0.00 ± 0.26	5	8.58 ± 0.51	−0.05 ± 0.45	0.78 ± 0.49	5	6.50 ± 0.08*	−2.50 ± 0.21*	−1.67 ± 0.27*	5
UCN1	10.02 ± 0.14	0.00 ± 0.20	0.00 ± 0.29	5	8.25 ± 0.20	0.00 ± 0.33	0.00 ± 0.39	5	8.33 ± 0.12	0.00 ± 0.17	0.00 ± 0.27	5
UCN2	9.50 ± 0.11*	−0.55 ± 0.19	0.00 ± 0.27	5	7.88 ± 0.41	−0.19 ± 0.42	0.36 ± 0.46	5	6.93 ± 0.06*	−1.42 ± 0.13*	−0.87 ± 0.23	5
UCN3	10.34 ± 0.11	0.34 ± 0.21	0.00 ± 0.29	5	9.18 ± 0.23	0.91 ± 0.38	0.57 ± 0.43	5	7.29 ± 0.06*	−1.09 ± 0.13*	−1.43 ± 0.25*	5

Note

Relative efficacy and signaling bias were calculated using UCN1 and cAMP as the reference agonist and signaling pathway. Data were analyzed using a student's *t* test (CRF_1_) or by one‐way ANOVA followed by a post‐hoc Dunnett's test (CRF_2_).

Data are mean ± SEM of the combined data from 5 independent experiments.

Abbeviations: CRF, corticotropin releasing factor; ERK1/2, extracellular signal‐regulated kinase 1/2; IP_1_, inositol monophosphate.

* *P* < .05 compared to UCN1.

In comparison to the CRF_1_ receptor, the CRF_2_ receptor displayed greater variation in ligand responses between pathways. CRF, UCN1, UCN2, and UCN3 all produced concentration‐dependent increases in cAMP, pERK1/2, and IP_1_ accumulation at this receptor (Figure 1D‐F; Table 1). The Hill slope was not significantly different from one when CRF, UCN1, UCN2, or UCN3 stimulated cAMP and IP_1_ accumulation_._ However, when pERK1/2 was measured, the Hill slope was 0.28, 0.51, 0.23, and 0.30 for CRF, UCN1, UCN2, and UCN3, respectively. Interestingly, the peptides were not equally active. UCN1 and UCN3 activated cAMP signaling more potently than UCN2 and CRF (Figure 1D; Table 1). In contrast, there was no significant difference in the ability of CRF, UCN1, UCN2, and UCN3 to produce pERK1/2, although UCN3 trended toward being the most potent (Figure 1E; Table 1). For IP_1_ accumulation, UCN1 was approximately 10‐, 25‐, and 67‐fold more potent than UCN3, UCN2, and CRF, respectively (Figure 1F; Table 1). CRF also displayed a lower *E*_max_ than the UCN peptides for the accumulation of IP_1_, suggesting that CRF is a partial agonist at this receptor via this pathway. These differences in signaling profiles were supported by analysis of biased signaling (Table 1), whereby relative to cAMP and pERK1/2, CRF, and UCN3 displayed lower potencies for the activation of IP_1_ signaling. This suggests that CRF and UCN3 are biased agonists relative to UCN1 with a preference for stimulating cAMP over IP_1_ accumulation (approximately 47‐ and 27‐fold, respectively).

### 3.2 Characterization of antagonist pharmacology at CRF receptors

Overall, the signaling behavior and identification of biased signaling for cAMP over IP_1_ accumulation by CRF and UCN3 relative to UCN1 at the CRF_2_ receptor indicated that the activation of these receptors is more complex than is currently appreciated. To further understand CRF receptor signaling behavior, the ability of antagonists to block CRF, and in some experiments, UCN1‐mediated cAMP and IP_1_ accumulation at CRF receptors were investigated. Three antagonists were selected; α‐helical CRF_(9‐41)_, astressin_2B_, and CP‐376,395.^40^, ^41^, ^42^ The majority of prior antagonist characterization has been conducted using competitive binding and IC_50_ format assays. Although these types of assays give a snapshot of antagonist activity, they do not have the depth of Schild analysis, which can highlight additional molecule behavior, such as partial agonism and insurmountable antagonism. Thus, where possible, we elected to undertake Schild‐style analysis.

### 3.3 α‐helical CRF_(9‐41)_ weakly discriminates between CRF receptors in transfected Cos7 cells

**FIGURE 2:**
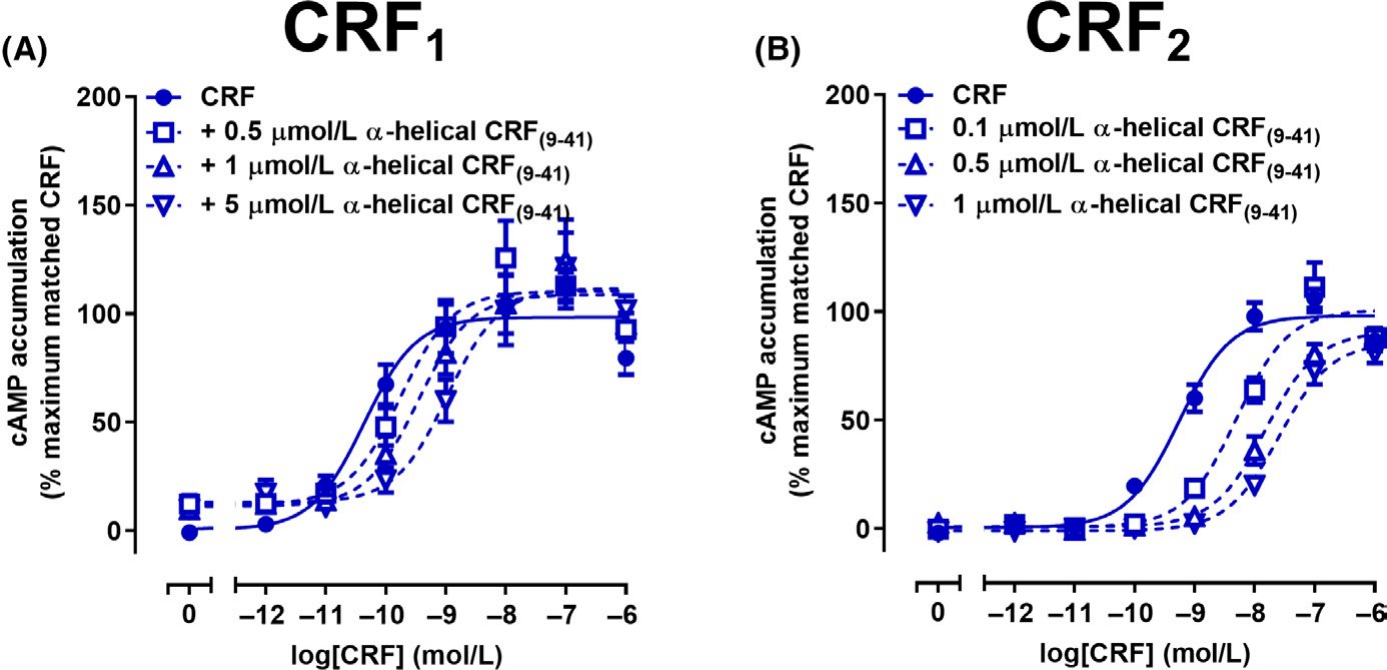
Antagonism of CRF‐mediated cAMP signaling by α‐helical CRF_(9‐41)_ in Cos7 cells expressing CRF_1_ or CRF_2_ receptors. A, Antagonism of cAMP accumulation by α‐helical CRF_(9‐41)_ at CRF_1_ receptors. B, Antagonism of cAMP accumulation by α‐helical CRF_(9‐41)_ at CRF_2_ receptors. Data points are the mean ± SEM of the combined data from five independent experiments, performed in triplicate. CRF, corticotropin releasing factor

α‐Helical CRF_(9‐41)_, has been reported as a competitive antagonist of CRF and UCN1 at both CRF_1_ and CRF_2_ receptors.^43^ CRF‐stimulated cAMP accumulation was antagonized by α‐helical CRF_(9‐41)_ at the CRF_1_ receptor (Figure 2A). Interestingly, α‐helical CRF_(9‐41)_ also weakly stimulated cAMP accumulation with an *E*_max_ of 14.8% indicating that it can act as a weak partial agonist of this receptor (Figure S3A). Similar partial agonism by α‐helical CRF_(9‐41)_ at the CRF_1_ receptor has previously been reported.^44^ Despite the elevation in basal cAMP with α‐helical CRF_(9‐41)_, global Schild analysis fitted the data well. The Schild slope was not significantly different from one and was therefore constrained to one. α‐helical CRF_(9‐41)_ antagonized CRF at the CRF_1_ receptor with a p*K_B_* of 6.77 (Table 2).

**TABLE 2:** Summary of antagonist potency (pK_B_) values in Cos7 cells transiently transfected with the CRF_1_ and CRF_2_ receptors

Peptide	cAMP						IP_1_	
	α‐Helical CRF_(9‐41)_	n	Astressin_2B_	n	CP‐376,395	n	CP‐376,395	n
hCRF_1α_ receptor								
CRF	6.77 ± 0.17	5	6.32 ± 0.13	5	6.90 ± 0.20*	5	7.55 ± 0.10*	5
UCN1	—		<5.3	3	5.32 ± 0.14	5	6.67 ± 0.08	5
hCRF_2α_ receptor								
CRF	7.73 ± 0.09	5	9.52 ± 0.16*	5	<4	5	NC	5
UCN1	—		8.44 ± 0.11	5	<4	5	3.40 ± 0.29	6

Note

Antagonist potency values (p*K_B_*) were determined using global Schild analysis for cAMP signaling or the Gaddum method for insurmountable antagonism for IP_1_ accumulation. Data were analyzed by a student's *t* test.

Data are mean ± SEM of the combined data from n independent experiments. NC; no curve could be fitted to the data.

Abbeviations: CRF, corticotropin releasing factor; IP_1_, inositol monophosphate.

* *P* < .05 compared to UCN1.

α‐Helical CRF_(9‐41)_ was approximately 10‐fold more potent at antagonizing CRF‐induced cAMP accumulation at the CRF_2_ receptor, compared to the CRF_1_ receptor (Figure 2B; Table 2). No partial agonism was observed for α‐helical CRF_(9‐41)_ at the CRF_2_ receptor (Figure S3B). The Schild slope was not significantly different to one and global Schild analysis indicated that α‐helical CRF_(9‐41)_ antagonized CRF at the CRF_2_ receptor with a p*K_B_* of 7.73 (Table 2).

### 3.4 Astressin_2B_ exhibits probe‐dependent antagonism at CRF receptors

**FIGURE 3:**
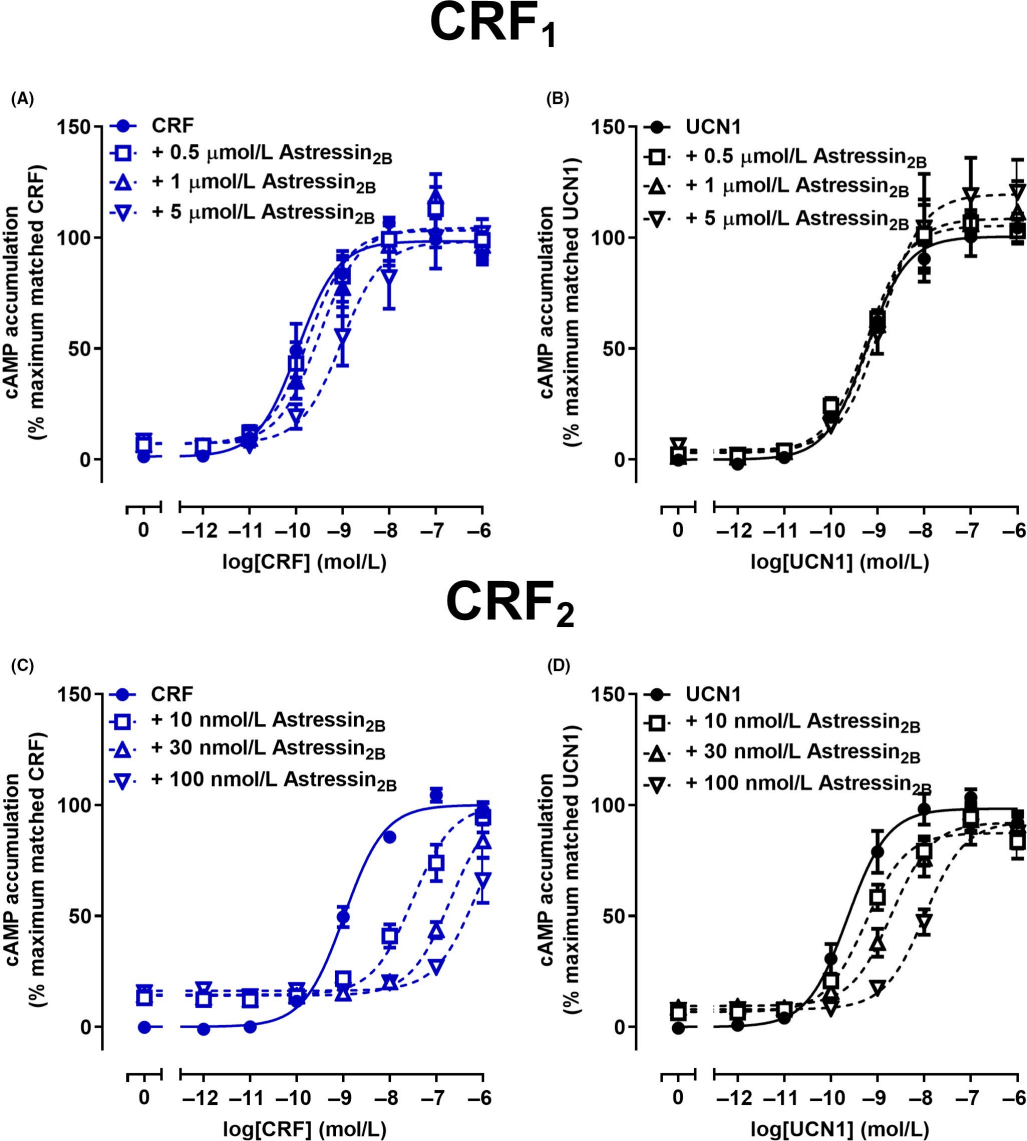
Antagonism of CRF or UCN1‐mediated cAMP signaling by astressin_2B_ in Cos7 cells expressing CRF_1_ or CRF_2_ receptors. A, Antagonism of CRF‐mediated cAMP accumulation by astressin_2B_ at CRF_1_ receptors. B, Antagonism of UCN1‐mediated cAMP accumulation by astressin_2B_ at CRF_1_ receptors. C, Antagonism of CRF‐mediated cAMP accumulation by astressin_2B_ at CRF_2_ receptors. D, Antagonism of UCN1‐mediated cAMP accumulation by astressin_2B_ at CRF_2_ receptors. Data points are the mean ± SEM of the combined data from 5 (A, C and D) or 3 (B) independent experiments, performed in triplicate. CRF, corticotropin releasing factor

Astressin_2B_, is a highly modified truncated peptide, which is reported to be a selective antagonist of the CRF_2_ receptor.^41^ Interestingly, CRF‐mediated cAMP signaling was antagonized by astressin_2B_ at the CRF_1_ receptor (Figure 3A). Global Schild analysis reflected this with a p*K_B_* of 6.32 (Table 2). However, concentrations of up to 5 μmol/L astressin_2B_ did not measurably antagonize UCN1‐mediated cAMP signaling at the CRF_1_ receptor (p*K_B_* reported as <5.3) (Figure 3B; Table 2).

Astressin_2B_ was at least 1000‐fold more potent at antagonizing either CRF or UCN1‐induced cAMP signaling at the CRF_2_ receptor, compared to the CRF_1_ receptor (Figure 3; Table 2). However, astressin_2B_ also acted as a weak partial agonist of the CRF_2_ receptor, stimulating cAMP accumulation with an *E*_max_ of 11.0%; this was not the case at the CRF_1_ receptor (Figure S4A,B). Global Schild analysis indicated that astressin_2B_ was approximately 10‐fold more potent at antagonizing CRF‐mediated (p*K_B_* of 9.52) than UCN1‐mediated (p*K_B_* of 8.44) cAMP signaling (Figure 3C,D; Table 2). These findings suggest that astressin_2B_ behaves as an agonist‐ or probe‐dependent antagonist at the CRF receptors, favoring the antagonism of CRF over UCN1‐mediated cAMP signaling.

### 3.5 CP‐376,395 exhibits probe‐dependent antagonism at the CRF_1_ receptor

**FIGURE 4:**
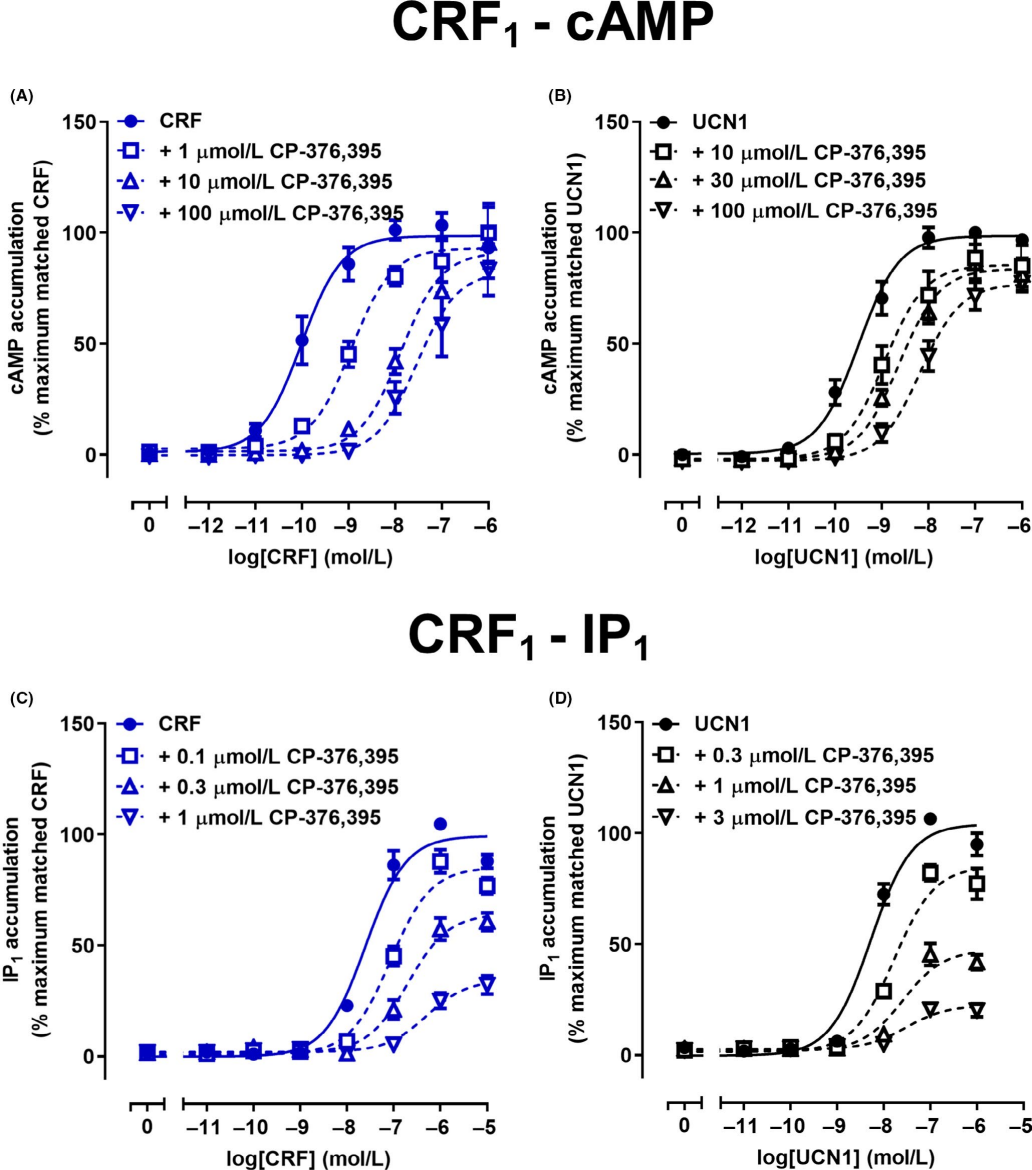
Antagonism of CRF or UCN1‐mediated cAMP and IP_1_ signaling by CP‐376,395 in Cos7 cells expressing CRF_1_ receptors. A, Antagonism of CRF‐mediated cAMP accumulation by CP‐376,395 at CRF_1_ receptors. B, Antagonism of UCN1‐mediated cAMP accumulation by CP‐376,395 at CRF_1_ receptors. C, Antagonism of CRF‐mediated IP_1_ accumulation by CP‐376,395 at CRF_1_ receptors. D, Antagonism of UCN1‐mediated IP_1_ accumulation by CP‐376,395 at CRF_1_ receptors. Data points are the mean ± SEM of the combined data from five independent experiments, performed in triplicate. CRF, corticotropin releasing factor; IP_1_, inositol monophosphate

CP‐376,395 is a small molecule antagonist reported to be selective for the CRF_1_ receptor.^42^ In contrast to α‐helical CRF_(9‐41)_ and astressin_2B_, which are larger peptide antagonists, CP‐376,395 displayed no evidence of partial agonism. CP‐376,395 effectively antagonized both CRF and UCN1‐mediated cAMP accumulation at the CRF_1_ receptor (Figure 4A,B; Table 2). Global Schild analysis indicated that CP‐376,395 was approximately 50‐fold more potent at antagonizing CRF‐mediated (p*K_B_* of 6.99) than UCN1‐mediated (p*K_B_* of 5.34) cAMP accumulation (Table 2). Interestingly, this finding suggests that CP‐376,395 behaves as an agonist‐ or probe‐dependent antagonist, favoring the antagonism of CRF over UCN1‐mediated cAMP accumulation. CP‐376,395 was used to stabilize a CRF_1_ receptor crystal structure.^45^ The structure suggests that CP‐376,395 binds at an allosteric site and may thus act as an allosteric modulator. Therefore, the data were also fitted to the operational model of allosterism. Probe‐dependent effects of CP‐376,395 were observed for antagonism of cAMP accumulation, where CP‐376,395 had a greater allosteric effect on CRF (*α* of 0.005) than UCN1 (*α* of 0.03) activity. The allosteric model suggested that CP‐376,395 had a small effect on agonist efficacy for CRF (*β* of 0.63) and UCN1 (*β* of 0.63). Conversely, *F* tests performed on the non‐linear fits indicated that there was no significant difference in *E*_max_ values. Antagonism of CRF and UCN1‐mediated IP_1_ accumulation was also examined at the CRF_1_ receptor (Figure 4C,D; Table 2). *F* tests conducted on individual data sets suggested a reduction in *E*_max_, indicative of a non‐competitive antagonist. To confirm that this was not a non‐specific effect on this pathway, the ability of 100 µmol/L CP‐376,395 to antagonize IP_1_ accumulation at the calcitonin receptor was tested (Figure S5). CP‐376,395 had no effect on IP_1_ accumulation at the calcitonin receptor, suggesting that the effects of CP‐376,395 on *E*_max_ were CRF_1_ receptor‐dependent. The reduction in *E*_max_ indicated that global Schild analysis was not an appropriate method to analyze antagonism, therefore, the method of Gaddum was used to determine antagonist potency for a non‐competitive or insurmountable antagonist.^34^ This suggested that CP‐376,395 was approximately 8‐fold more potent at antagonizing CRF‐mediated (p*K_B_* of 7.55) than UCN1‐mediated (p*K_B_* of 6.67) IP_1_ accumulation at the CRF_1_ receptor (Table 2). Although, the method of Gaddum for an insurmountable antagonist fitted the data well, this analysis does not consider the possible allosteric nature of CP‐376,395 action. Therefore the combined data were also fitted using the operational model of allosterism. This confirmed the probe‐dependence of CP‐376,395 antagonism of IP_1_ accumulation at the CRF_1_ receptor. A greater allosteric effect of CP‐376,395 was observed for CRF (*α* of 0.03) compared to UCN1 (*α* of 0.15) activity. However due to apparent high negative co‐operatively, *β* values could not be determined for the effect of CP‐376,395 on either CRF‐ and UCN1‐mediated IP_1_ accumulation and were assumed to be ~0.

**FIGURE 5:**
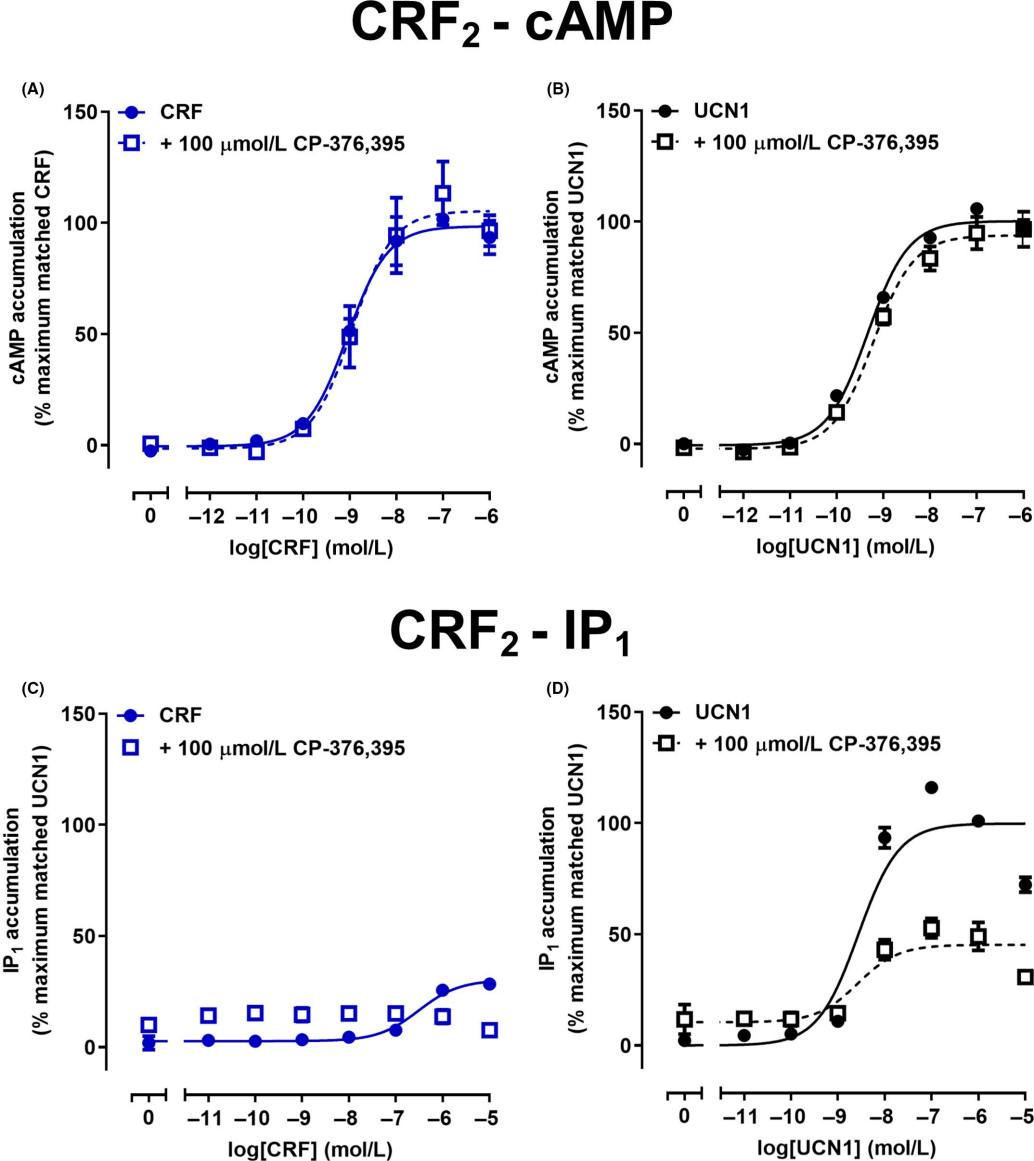
Antagonism of CRF or UCN1‐mediated cAMP and IP_1_ signaling by CP‐376,395 in Cos7 cells expressing CRF_2_ receptors. A, Antagonism of CRF‐mediated cAMP accumulation by CP‐376,395 at CRF_2_ receptors. B, Antagonism of UCN1‐mediated cAMP accumulation by CP‐376,395 at CRF_2_ receptors. C, Antagonism of CRF‐mediated IP_1_ accumulation by CP‐376,395 at CRF_2_ receptors. D, Antagonism of UCN1‐mediated IP_1_ accumulation by CP‐376,395 at CRF_2_ receptors. Data points are the mean ± SEM of the combined data from 5 (A‐C) or 6 (D) independent experiments, performed in triplicate. CRF, corticotropin releasing factor; IP_1_, inositol monophosphate

To confirm the specificity of CP‐376,395 for the CRF_1_ receptor, antagonist activity was compared at the CRF_2_ receptor. One hundred micromolar CP‐376,395 had no effect on either CRF or UCN1‐mediated cAMP accumulation at the CRF_2_ receptor (Figure 5A,B; Table 2). This suggests that CP‐376,395 had a p*K_B_* of <4 at the CRF_2_ receptor and therefore was at least 800 or 20‐fold more potent at antagonizing either CRF or UCN1‐induced cAMP signaling at the CRF_1_ receptor compared to the CRF_2_ receptor. To further characterize the properties of CP‐376,395, antagonism of CRF and UCN1‐mediated IP_1_ accumulation was examined at the CRF_2_ receptor (Figure 5C,D; Table 2). Antagonism of CRF‐mediated IP_1_ accumulation could not be quantified due to weak CRF‐mediated IP_1_ accumulation, although the response appeared to be abolished in the presence of 100 µmol/L CP‐376,395. At the CRF_2_ receptor, *F* tests conducted on individual data sets suggested a reduction in *E*_max_ indicative of a non‐competitive or insurmountable antagonist. The method of Gaddum for a non‐competitive or insurmountable antagonist^34^ suggested that CP‐376,395 antagonized UCN1‐mediated IP_1_ accumulation with a p*K_B_* of 3.40 (Table 2).

### 3.6 CRF receptors do not increase RAMP expression at the cell surface

**FIGURE 6:**
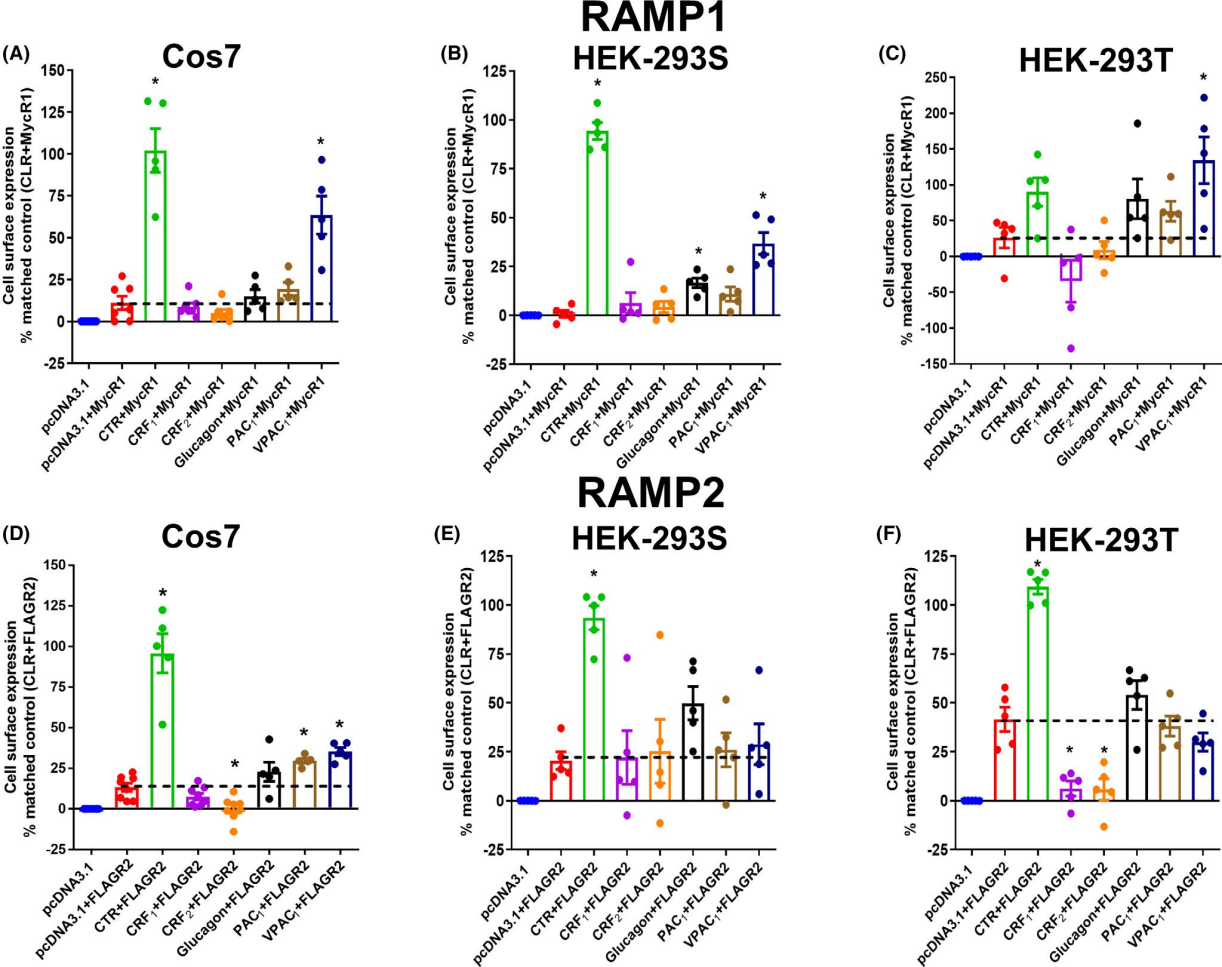
Effect of CLR, CTR, CRF_1_, CRF_2_, Glucagon, PAC_1_, and VPAC_1_ receptors on the cell surface expression of myc‐tagged RAMP1 (MycR1) and FLAG‐tagged RAMP2 (FLAGR2) in Cos7, HEK‐293S, and HEK‐293T cells. A, Cell surface expression of myc‐RAMP1 in Cos7 cells. B, Cell surface expression of myc‐RAMP1 in HEK‐293 cells. C, Cell surface expression of myc‐RAMP1 in HEK‐293T cells. D, Cell surface expression of FLAG‐RAMP2 in Cos7 cells. E, Cell surface expression of FLAG‐RAMP2 in HEK‐293S cells. F, Cell surface expression of FLAG‐RAMP2 in HEK‐293T cells. The dashed line represents the level of RAMP expression at the cell surface in the absence of co‐transfected receptor. Data were analyzed by one‐way ANOVA followed by a post hoc Dunnett's test. *P < .05. Data points are the mean ± SEM of the combined data from five independent experiments, performed in quadruplicate. CLR, calcitonin receptor‐like receptor; CRF, corticotropin releasing factor; RAMP, receptor activity‐modifying protein

**FIGURE 7:**
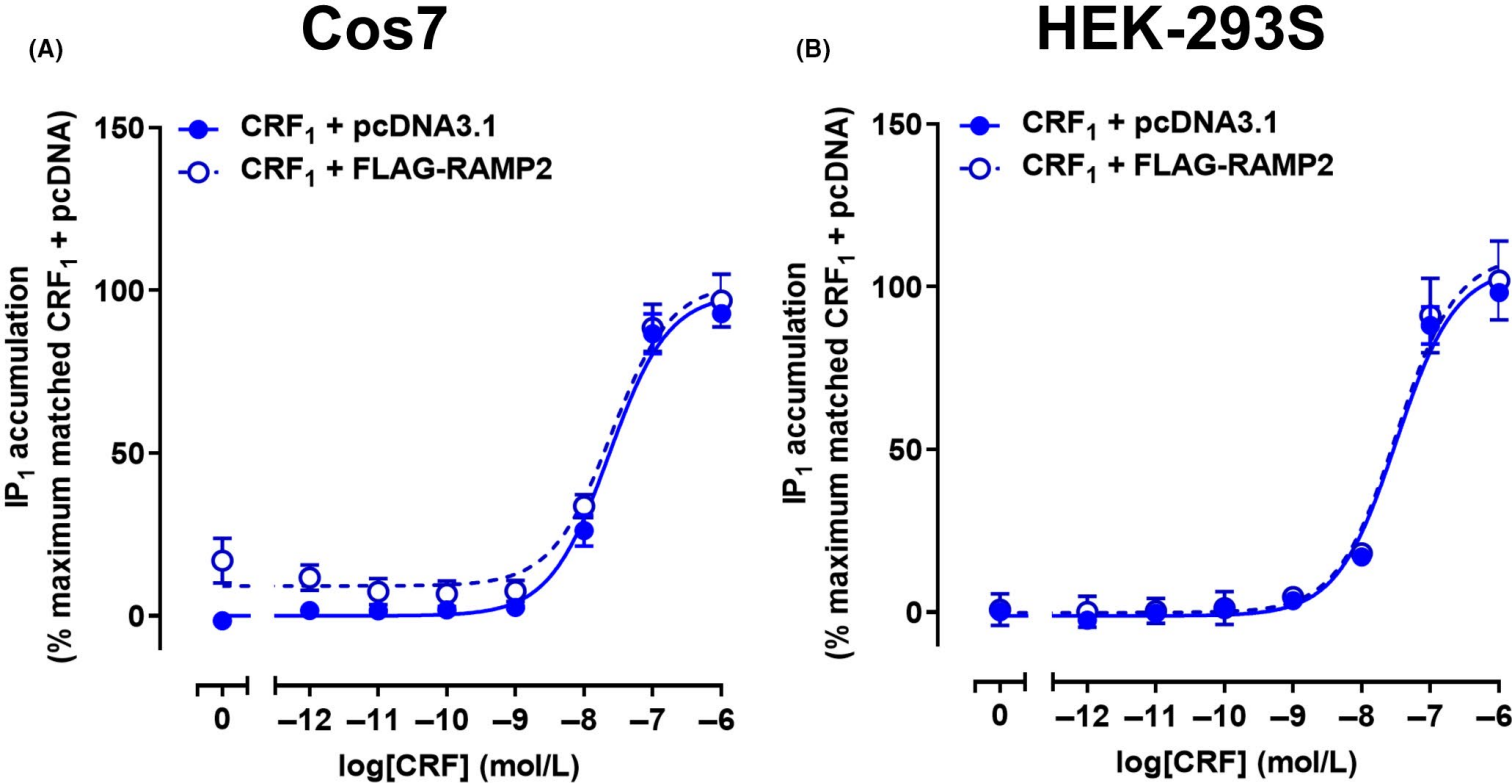
Effect of RAMP2 on CRF‐mediated IP_1_ accumulation in Cos7 and HEK‐293S cells expressing CRF_1_ receptors. A, Stimulation of IP_1_ accumulation by CRF in Cos7 cells transfected with CRF_2_ receptors and pcDNA3.1 or FLAG‐RAMP2. B, Stimulation of IP_1_ accumulation by CRF in HEK‐293S cells transfected with CRF_1_ receptors and pcDNA3.1 or FLAG‐RAMP2. Data points are the mean ± SEM of the combined data from 4 (A) or 3 (B) independent experiments, performed in triplicate. CRF, corticotropin releasing factor; IP_1_, inositol monophosphate

Previous research has suggested that both the CRF_1α_ and CRF_1β_ receptor splice variants can interact with RAMP2, as determined by enhancement of RAMP2 cell surface expression.^19^, ^21^ RAMP2 was also reported to increase Gαq‐coupling of the CRF_1β_ receptor variant.^19^ We hypothesized that interactions between the CRF receptors and RAMPs could further alter signaling and antagonist behavior. To address this question, we first sought to confirm that the CRF_1_ receptor can affect RAMP2 surface expression, and to compare this to the CRF_2_ receptor. Two robust RAMP partners—CLR and CTR were used as positive controls, and additional class B GPCRs (glucagon, PAC_1_, and VPAC_1_ receptors) were also examined in parallel. In Cos7 cells, the cell surface expression of myc‐tagged RAMP1 was significantly increased in the presence of the CLR, CTR, and VPAC_1_ receptors. However, no change in RAMP1 surface expression was observed with CRF_1_ and CRF_2_ receptors (Figure 6A). Similar results were observed in HEK‐293S cells (Figure 6B). Only VPAC_1_ displayed a significant increase in RAMP1 surface expression in HEK‐293T cells (Figure 6C). In Cos7 cells, CLR, CTR, PAC_1_, and VPAC_1_ significantly increased FLAG‐RAMP2 cell surface expression. However, no change in RAMP2 surface expression was observed with the CRF_1_ receptor and the CRF_2_ receptor resulted in a slight decrease in cell surface expression (Figure 6D). In HEK‐293S and HEK‐293T cells only CLR and CTR significantly increased FLAG‐RAMP2 cell surface expression (Figure 6E,F). Interestingly, in HEK‐293T cells both CRF_1_ and CRF_2_ decreased FLAG‐RAMP2 cell surface expression (Figure 6F). To determine the effect of CRF receptors on RAMP3 cell surface expression we examined two different tagged constructs; FLAG‐RAMP3 and myc‐RAMP3. FLAG‐RAMP3 and myc‐RAMP3 did not display normal function and therefore could not be used (Figure S6). To confirm that any interactions between the CRF_1_ receptor and RAMP2 were not missed, we assessed IP_1_ accumulation in Cos7 and HEK‐293S cells in the absence and presence of RAMP2 (Figure 7A,B). No difference in the maximal IP_1_ response was observed in either Cos7 or HEK‐293S cells. Overall, these data suggested that in our hands, there was no clear effect of either CRF receptor on RAMP1 or 2 cell surface expression, nor of RAMP2 on CRF_1_ receptor IP_1_ accumulation. Therefore, no further RAMP experiments were conducted.

## 4 DISCUSSION AND CONCLUSIONS

The CRF receptors have been the target of intensive efforts to develop new drugs, with the major clinical focus on the treatment of anxiety, depression, and drug‐dependence.^18^ Although no specific CRF receptor targeted therapy has been approved by regulatory authorities, several small molecule antagonists have been developed and there is considerable data around the safety and pharmacokinetics of these molecules.^18^, ^42^ This wealth of information makes the CRF receptors a tantalizing target for the development of new therapeutics for other disorders. It may be possible to fast‐track a new therapeutic into the clinic by building on existing knowledge. However, in order to achieve this, we first need a better understanding of how the CRF receptors signal and how effectively CRF receptor antagonists block signaling.

The presence of multiple endogenous ligands in the CRF peptide family which are capable of binding to the same receptors, likely provides significant redundancy in the system. This also suggests that endogenous biased signaling may be an important contributor to different biological activities reported for CRF and the UCN peptides. Furthermore, biased signaling has been observed for other closely related class B GPCRs; including the GLP‐1 and PAC_1_ receptors.^29^, ^46^, ^47^, ^48^ To investigate this possibility we examined the ability of species‐matched CRF receptors and agonists to activate cAMP, pERK1/2, and IP_1_ accumulation and calculated bias for these agonists.^33^ Overall, the CRF receptors displayed similar agonist pharmacology to that described in the literature.^49^ However, we observed some subtle differences between signaling pathways with magnitudes similar to those reported at related receptors. Specifically, CRF displayed biased signaling towards accumulation of cAMP over IP_1_ relative to UCN1 at the CRF_1_ receptor. Furthermore, at the CRF_2_ receptor both CRF and UCN3 displayed biased signaling relative to UCN1 for cAMP signaling over IP_1_. This suggests that CRF and UCN3 are biased agonists for Gαs protein‐coupled signaling relative to UCN1. UCN2 displays similar behavior to UCN1 at the CRF_2_ receptor. Given the extensive study of the CRF receptors, it is unsurprising that there are hints of similar signaling bias in the literature that we now more formally describe. For example, there appears to be similar bias for cAMP signaling over Ca^2+^ mobilization using ovine CRF and human UCN3 relative to other agonists in HEK‐293 cells stably transfected with the hCRF_2α_ receptor.^50^ These similarities suggest that cAMP signaling bias for CRF and UCN relative to other agonists at the CRF_2_ receptor may be widespread among different cell types. This phenomenon may be particularly relevant for future drug discovery efforts, given the importance cAMP signaling in CRF action and the reported role of cAMP signaling the pathology of several disorders.^38^, ^51^, ^52^ However, biased signaling could be masked by the differential expression of receptors or signaling proteins. For instance, SK‐N‐MC cells did not display Ca^2+^ mobilization in response to CRF_1_ or CRF_2_ receptor activation, presumably because they lack the proteins required to signal by this pathway.^50^ Due to the similarities we observed between the behavior of cAMP and pERK1/2 signaling it is tempting to suggest that pERK1/2 is downstream of Gαs and cAMP. However, the activation of pERK1/2 has been reported downstream of both Gαs and Gαq in several studies.^52^, ^53^, ^54^, ^55^, ^56^ Interestingly in the current study, the Hill slope of the pERK1/2 agonist curves did not equal one. This suggests the phosphorylation of ERK1/2 may be due to multiple agonist binding sites or was downstream of multiple signaling events. It is therefore conceivable that there is a biased component of pERK1/2 signaling, which may be masked by the cAMP signaling response and the inherent variability of the data. Furthermore, as changes in individual signaling molecules, including cAMP, pERK1/2 and IP_1,_ can be downstream of multiple effectors, it is possible that biased signaling is being underestimated or masked by opposing signaling cascades. The role biased agonists may play endogenously in CRF receptor signaling is not clear, but could partly explain subtle differences observed between the biology of UCN2 and UCN3.^57^ However, the precise CRF receptor splice variant and peptide expressed at a given site of action will also dictate the biological outcomes. More research is clearly required to understand this complex receptor signaling system.

Profiling antagonist activity at the CRF_1_ and CRF_2_ receptors revealed several interesting findings, including partial agonism, apparent agonist (probe)‐dependent antagonism and apparent pathway‐dependent non‐competitive antagonism or negative allosteric modulation. The peptide antagonists, α‐helical CRF_(9‐41)_ and astressin_2B_, displayed similar antagonist potency to previous reports.^44^, ^58^, ^59^ However, both α‐helical CRF_(9‐41)_ and astressin_2B_ displayed weak partial agonism at the CRF_1_ and the CRF_2_ receptors, respectively. This phenomenon may have resulted in the antagonist determination being inaccurate and potentially over‐estimated. The appearance of partial agonism is perhaps unsurprising as several antagonists derived by truncating the endogenous peptide for class B GPCRs display this property and partial agonism has previously been reported for α‐helical CRF_(9‐41)_ in a receptor dependent manner.^29^, ^31^, ^44^, ^60^ However, in other studies partial agonism is either not observed or reported.^58^, ^61^ This inconsistency may reflect the difficulty in detecting a weak agonist response, differences in receptor expression or batch‐dependent variation in the antagonist preparations. Thus, caution should be exercised when using peptide antagonists, particularly in vivo where the administered doses used may be limited and multiple receptor subtypes may be present.

In this research, CRF and UCN1 were both utilized to define the antagonist properties of astressin_2B_ and CP‐376,395. The results indicated that UCN1 was antagonized less potently when compared to CRF. This relationship was evident for the CRF_1_ receptor, however, at the CRF_2_ receptor the weaker potency of CRF and CP‐376,395 resulted in this being difficult to confirm. Most prior studies have used binding assays or functional IC_50_ style approaches and typically a single agonist to define antagonism and therefore may not have been able to detect such a difference. However, this apparent agonist‐dependent antagonism may have profound implications for drug‐discovery. If complete blockade of UCN1 activity is required for full efficacy of an antagonist drug, then CRF would not be the appropriate agonist to use in screening campaigns as this would over‐estimate effectiveness. Furthermore, this observation may open up opportunities to develop antagonists capable of specifically blocking a CRF receptor mediated physiological response due to one peptide, without altering physiological responses caused by other ligands. Agonist‐dependent effects should be carefully considered for future studies of CRF receptors.

Non‐competitive or insurmountable antagonist‐like behaviors which display a reduction in *E*_max_, have been reported for several small molecule CRF_1_ receptor antagonists.^62^, ^63^ As speculated for CP‐376,395, these molecules may be acting, and therefore better described, as negative allosteric modulators.^45^ However, given the relatively large area involved in endogenous agonist binding to a class B GPCR and the potential for small molecules to engage with a GPCR as several different sites, further investigation, such as receptor mutagenesis is required to confirm that the activity of CP‐376,395 and related compounds occurs at an allosteric site.^64^ At the CRF_1_ receptor, non‐competitive antagonism for the blockade of cAMP accumulation is reportedly directly correlated with a slow dissociation or off‐rate for small molecule antagonists.^63^ Interestingly, this differed from those observed in the current study. Here, CP‐376,395 acted as a competitive antagonist of cAMP accumulation, but a non‐competitive antagonist of IP_1_ accumulation. We cannot rule out the possibility that this discrepancy is due to CP‐376,395 not reaching binding equilibrium during the time course of the cAMP assay, although a preliminary cAMP antagonism experiment suggested that a 75 minute pre‐incubation did not increase antagonism or result in a reduction of *E*_max_. The differences in antagonist behavior between signaling pathways may be contributed to by the phenomenon of receptor reserve. Relatively high levels of receptor expression can mask a drop in *E*_max_ if maximum activity only requires a small proportion of receptors to be activated. It is possible that in transiently transfected Cos7 cells, CRF receptors have weaker efficacy for the activation of IP_1_ responses compared to cAMP, resulting in cAMP responses potentially being resistant to antagonists that reduce *E*_max._ It is interesting to note that a similar difference was observed for the blockade of UCN1, where antalarmin was a competitive antagonist of Gαs and a non‐competitive antagonist of Gαi activity.^58^ Whether receptor reserve is involved in these differences in antagonist behavior and how these findings translate to endogenously expressed receptor systems should be investigated further.

The most compelling data for RAMP‐GPCR interactions centers on the class B GPCRs; CLR and CTR. CLR is an obligate heterodimer, requiring RAMPs for function and both CLR and CTR have been co‐localized with RAMP1 in rat and human tissues.^65^, ^66^ However, few of the other reported RAMP‐GPCR interactions have been duplicated or validated in vivo.^23^ One of the more compelling interactions reported was between the CRF_1β_ receptor with RAMP2, which enhanced Gαq coupling, resulting in increased Ca^2+^ mobilization.^19^ This was followed‐up by a second study that showed both CRF_1α_ and CRF_1β_, but not CRF_2β_, interact with RAMP2 and was supported by prior in vivo data from RAMP2^−/−^ mouse models, which display a weaker plasma ACTH response to CRF.^19^, ^21^ Given the close evolutionary relationship between all class B GPCRs and specifically between the CRF receptors, we hypothesized that RAMPs may also interact with CRF_2α_ receptors. Surprisingly, in the current study, neither CRF_1α_ nor CRF_2α_ increased RAMP1 or RAMP2 cell surface expression in the three cell lines tested. However, the current study is somewhat in agreement with a recent report, where the CRF_1_ receptor only weakly interacted with RAMP2 and the CRF_2_ receptor did not interact with either RAMP1 or RAMP2.^22^ Experiments using RAMP3 were halted as neither construct was functional in our assays. We confirmed that the sequences of the FLAG‐RAMP3 were the same as has been reported previously.^19^, ^67^ There is no clear explanation for this difference. In contrast to the previous study, the presence of RAMP2 did not alter Gαq coupled CRF_1_ receptor signaling. The reasons for the discrepancy in the effect of CFR_1α_ on RAMP2 between studies is not clear. However, this may relate to differences between the expression level of RAMP2 or CFR_1α_ between studies and the capacity to detect weak or uncommon interactions. We also tested in parallel three other class B GPCRs that had previously been shown to interact with RAMPs; the glucagon, VPAC_1_ and PAC_1_ receptors.^19^, ^22^, ^68^ The results from these other receptors tested was also mixed. The PAC_1_ receptor, which is reported to interact with all three RAMPs,^22^ only translocated RAMP2 in Cos7, but not HEK‐293S or HEK‐293T cells in the current study, although the effect in Cos7 cells was very small. In one study, the VPAC_1_ receptor was reported to translocate all three RAMPs to the cell surface,^69^ however in a second study VPAC_1_ only interacted with RAMP2 or RAMP3.^22^ In the current study, we observed an increase in RAMP1 surface expression in all three cell lines, but only saw increased surface expression with RAMP2 in Cos7 cells. This suggests that for VAPC_1_ receptors, translocation of RAMP1 to the cell surface is more robust than for RAMP2. Inconsistencies between studies have also previously been reported for the glucagon receptor.^22^, ^67^, ^68^, ^69^ In contrast to initial studies where surface expression was increased, we observed no significant changes in RAMP2 cell surface expression when co‐expressed with the glucagon receptor.^69^ Similarly, two distinct studies reported that co‐expression of RAMP2 with the glucagon receptor had the opposite effects on cAMP production.^67^, ^68^ These differences between studies may simply reflect the difficulties associated with investigating non‐obligate heterodimers and that more sensitive methods may be required to detect subtle interactions between receptors and RAMPs. However, differences may also relate to the precise cellular content. For instance the relatively high expression of RAMP1 and RAMP2 reported in HEK‐293T cells may explain the different observations to Cos7 and HEK‐293S cells, which do not express RAMP1, RAMP2, or RAMP3.^28^, ^70^, ^71^ The variation in emerging data suggests that RAMP‐receptor interactions need careful validation and whilst a useful tool, cell surface translocation experiments are unlikely to be conclusive as a stand‐alone measure. In particular, over‐expression of receptors and RAMPs in heterologous systems may lead to false positive results as this may be sufficient to facilitate a normally unfavorable biological interaction. Similarly, receptors or RAMPs modified to contain epitope tags, fluorescent labels or for molecular complementation studies may have altered behavior. These types of effects have been observed with other types of GPCR dimerization studies, and includes “bystander” effects.^72^ This is a receptor nomenclature issue because the identification of a RAMP‐GPCR partner is in essence the identification of a novel receptor subtype. Thus, multiple independent studies should draw similar conclusions before the complexes can be ratified as genuine novel receptors.

There is significant unmet clinical need in the treatment of stress and anxiety. Despite setbacks, the CRF receptors remain a tantalizing target for the development of new therapeutics for stress and anxiety and may have utility in other disorders, due to the wealth of information that exists. Our observations of biased agonism and agonist‐dependent antagonism illustrate some of the complexity involved in understanding the CRF receptor family and offer new avenues for developing drugs. New medicines could be tailored to activate a specific signaling pathway or block a specific agonist through a CRF receptor. Based on our findings we propose that the already complex pharmacology associated with the CRF receptors may be underappreciated and requires further investigation.

### 4.1 Nomenclature of targets and ligands

Key protein targets and ligands in this article are hyperlinked to corresponding entries in http://www.guidetopharmacology.org, the common portal for data from the IUPHAR/BPS Guide to PHARMACOLOGY,^73^ and are permanently archived in the Concise Guide to PHARMACOLOGY 2017.^40^

## CONFLICT OF INTEREST

The authors declare no conflicts of interest.

## AUTHOR’S CONTRIBUTIONS

The study was conceived and designed by CSW and DLH. ZT designed, performed the majority of experiments, and analyzed the data. PW and CSW performed some antagonist experiments. CSW, DLH and ZT drafted the manuscript. All authors approved the manuscript.

## Supporting information

Fig S1‐S6
