# Supplementary material for: Agonist bias and agonist‐dependent antagonism at corticotrophin releasing factor receptors
Source: Pharmacol Res Perspect. 2020 Jun 11;8(3):e00595. doi: 10.1002/prp2.595 (PMC7290078; doi:10.1002/prp2.595)
Supplement: Supplementary file 1 — Fig S1‐S6 [file PRP2-8-e00595-s001.pdf]

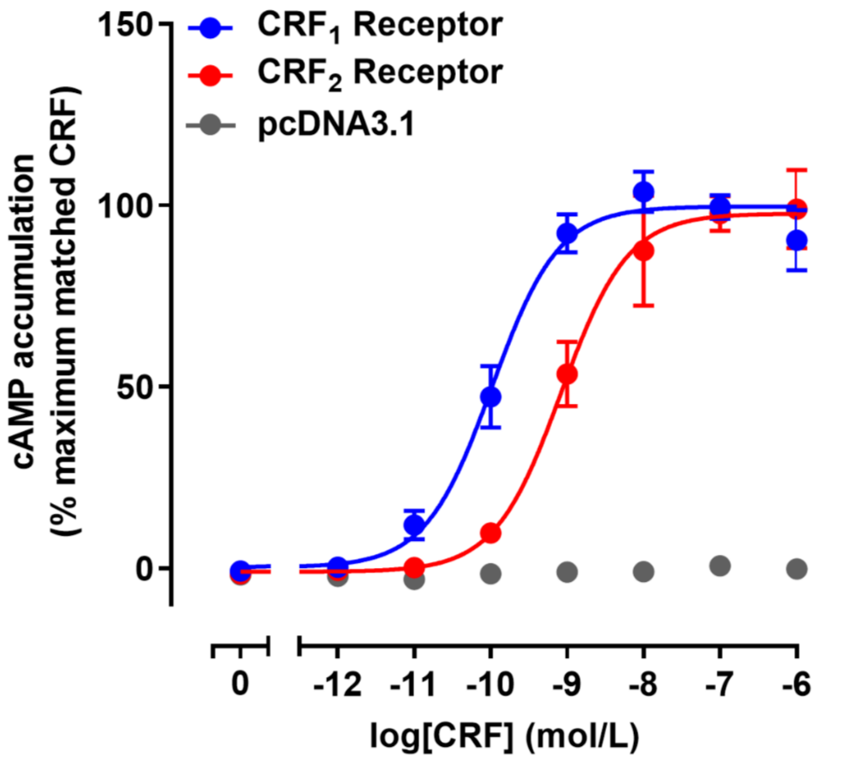

Figure S1. Stimulation of cAMP signalling by CRF in Cos7 cells transfected with CRF_1, CRF_2 or pcDNA3.1. Data points are the mean ± SEM of the combined data from 3 independent experiments, performed in triplicate.

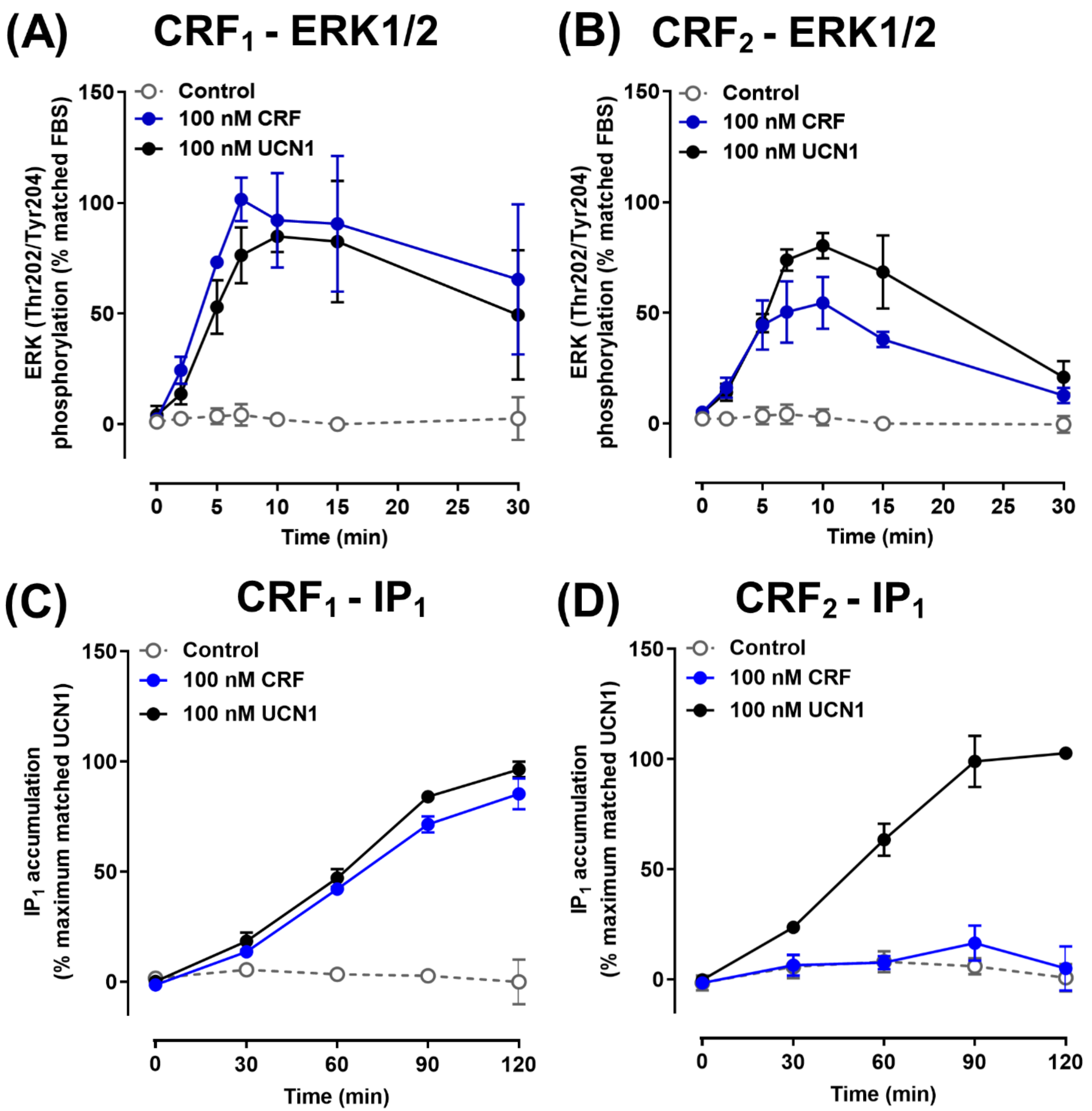

Figure S2. Time-courses of CRF-mediated ERK1/2 phosphorylation and IP_1 accumulation in Cos7 cells expressing CRF_1 or CRF_2 receptors. (A) Stimulation of ERK1/2 phosphorylation at CRF_1 receptors. (B) Stimulation of IP_1 accumulation at CRF_1 receptors. (C) Stimulation of ERK1/2 phosphorylation at CRF_2 receptors. (B) Stimulation of IP_1 accumulation at CRF_2 receptors. Data points are the mean ± SEM of the combined data from 3 independent experiments, performed in triplicate.

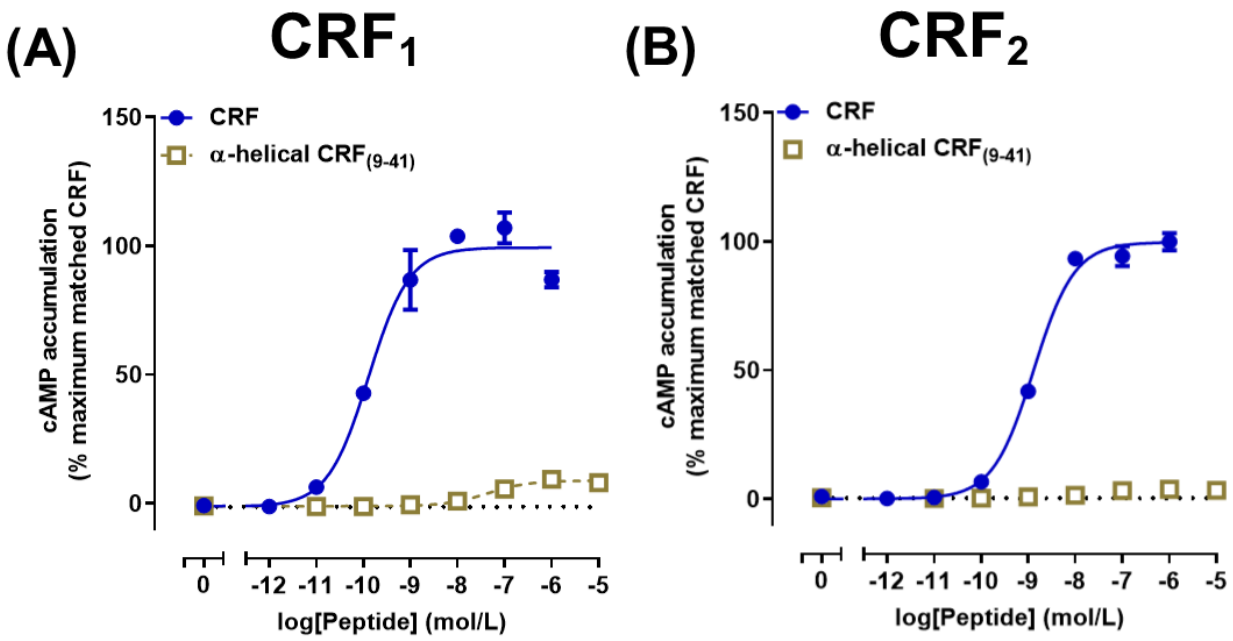

Figure S3. Stimulation of cAMP signalling by CRF and α-helical $CRF_{(9\text{-}41)}$ in Cos7 cells transfected with CRF_1 or CRF_2 receptors. (A) Stimulation of cAMP accumulation by CRF and α-helical $CRF_{(9\text{-}41)}$ at CRF_1 receptors. (B) Stimulation of cAMP accumulation by CRF and α-helical $CRF_{(9\text{-}41)}$ at CRF_2 receptors. Data points are the mean ± SEM of the combined data from 3 independent experiments, performed in triplicate.

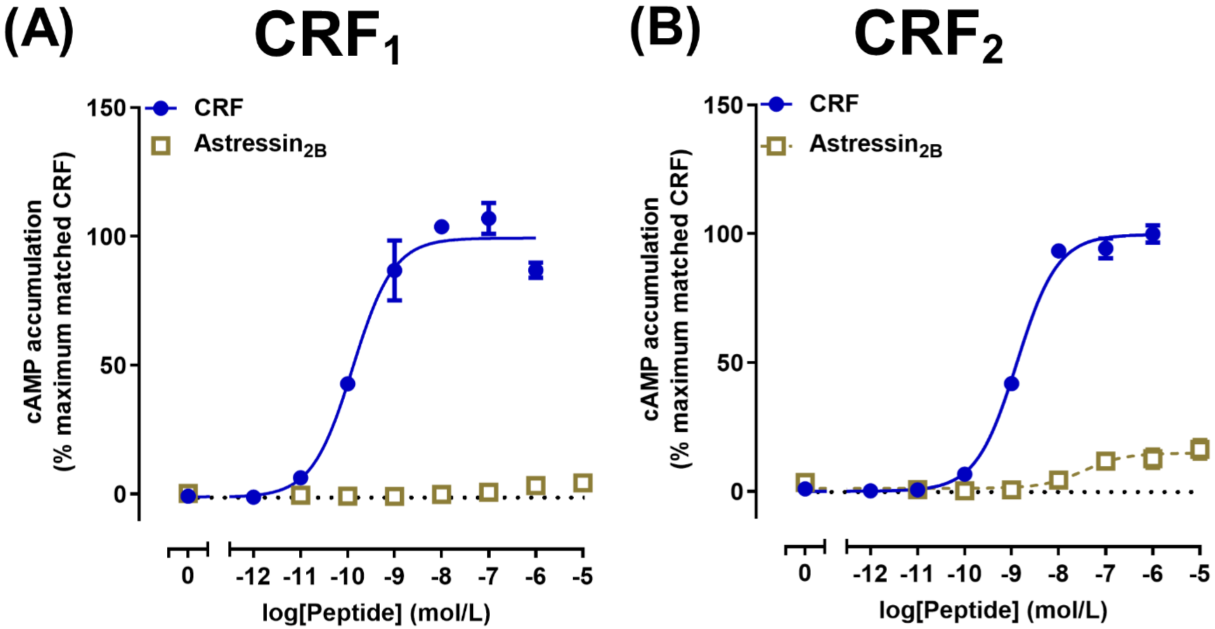

Figure S4. Stimulation of cAMP signalling by CRF and astressin$_{2B}$ in Cos7 cells transfected with CRF$_1$ or CRF$_2$ receptors. (A) Stimulation of cAMP accumulation by CRF and astressin$_{2B}$ at CRF$_1$ receptors. (B) Stimulation of cAMP accumulation by CRF and astressin$_{2B}$ at CRF$_2$ receptors. Data points are the mean ± SEM of the combined data from 3 independent experiments, performed in triplicate.

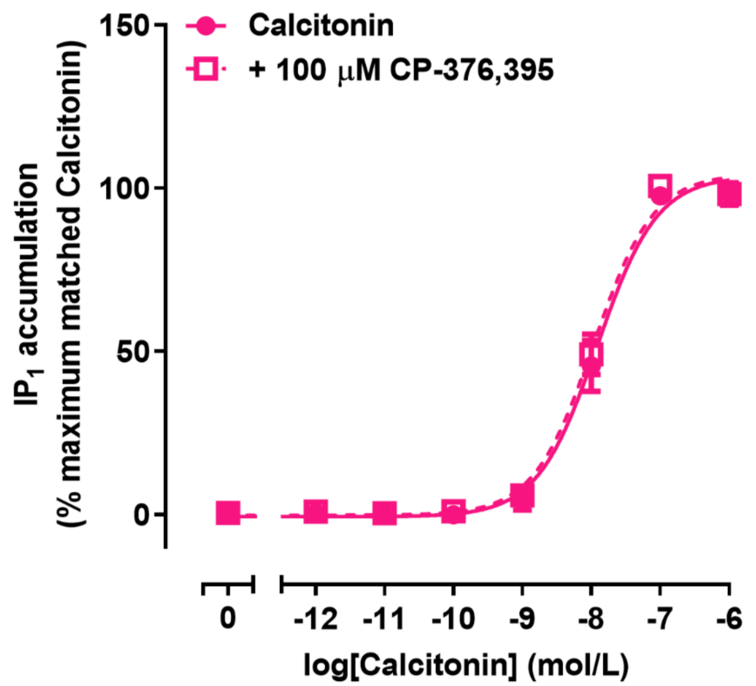

Figure S5. Antagonism of calcitonin-mediated IP_1 accumulation by 100 µM CP-376,395 in Cos7 cells expressing the human calcitonin receptor. Data points are the mean ± SEM of the combined data from 3 independent experiments, performed in triplicate.

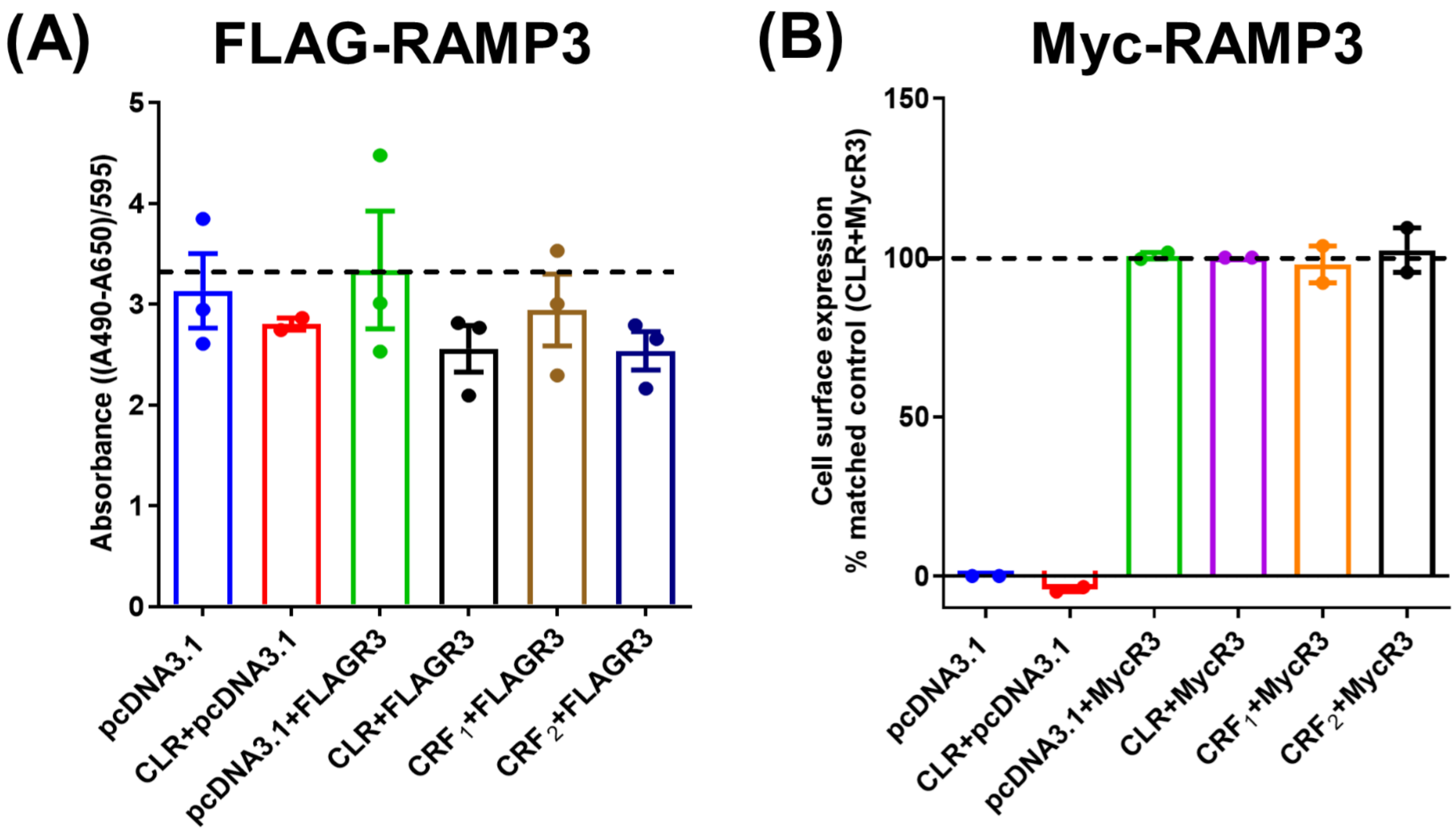

Figure S6. Effect of CLR, CRF_1 and CRF_2 receptors on the cell surface expression of FLAG-tagged RAMP3 (FLAGR3) and myc-tagged RAMP3 (MycR3) in Cos7 cells. (A) Cell surface expression of FLAG-RAMP3 in Cos7 cells. (B) Cell surface expression of myc-RAMP3 in Cos7 cells. The dashed line represents the level of RAMP expression at the cell surface in the absence of co-transfected receptor. Data are mean ± SEM of triplicates from a single representative experiment, which was performed two (Myc-RAMP3) or three (FLAG-RAMP3) times.
